# Supplementary material for: Simultaneous measurement of excitation-contraction coupling parameters identifies mechanisms underlying contractile responses of hiPSC-derived cardiomyocytes
Source: Nat Commun. 2019 Sep 20;10:4325. doi: 10.1038/s41467-019-12354-8 (PMC6754438; doi:10.1038/s41467-019-12354-8)
Supplement: Supplementary file 1 — Supplementary Information [file 41467_2019_12354_MOESM1_ESM.pdf]

Simultaneous measurement of excitation-contraction coupling parameters identifies mechanisms underlying contractile responses of hiPSC-derived cardiomyocytes

Van Meer et al.

Supplementary Information

SUPPLEMENTARY FIGURES

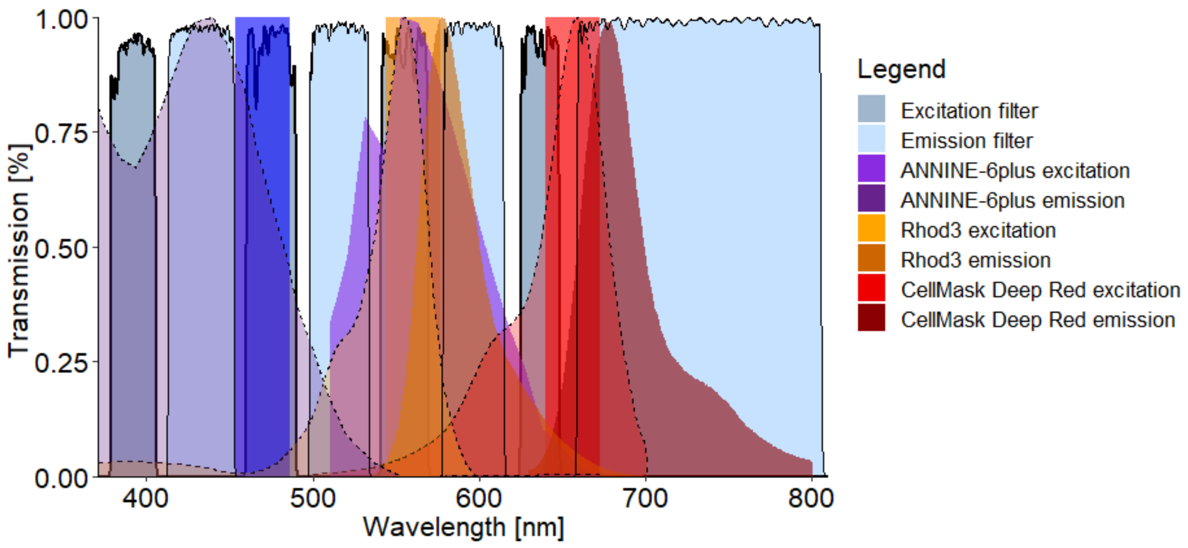

Supplemantary Figure 1: Spectral overview of TTM system and dyes used.

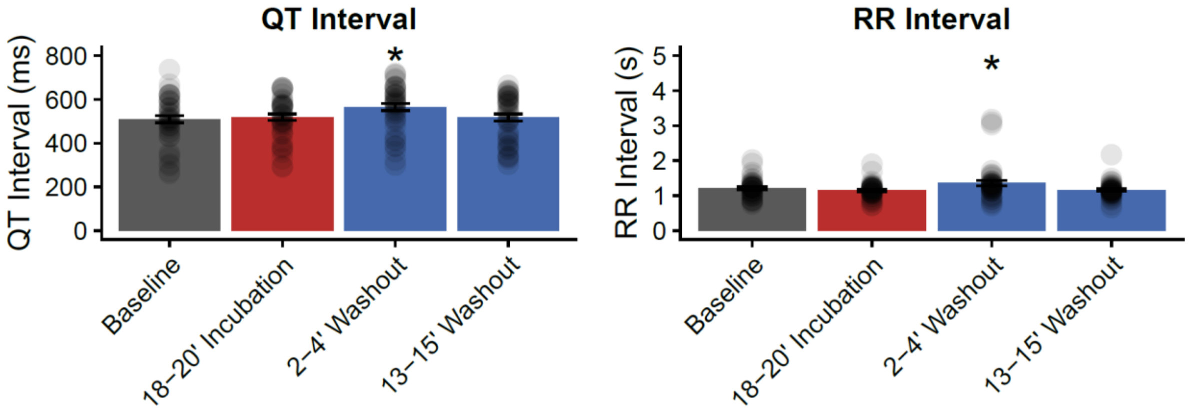

Supplemantary Figure 2: Effect on the electrophysiological properties of hiPSC-CMs by combined loading of ANNINE-6plus, Rhod-3 and CellMask Deep Red. QT and RR-interval are shown before addition (grey), after 18-20 minutes incubation (red) and 2-4 and 13-15 minutes after washout (blue). N=46 measurements. One-way ANOVA was used to test for differences in means between conditions but all p-values were larger than 0.05. The bars in the bar graph indicate the mean and the error bars indicate SEM. Source data are provided as a Source Data file.

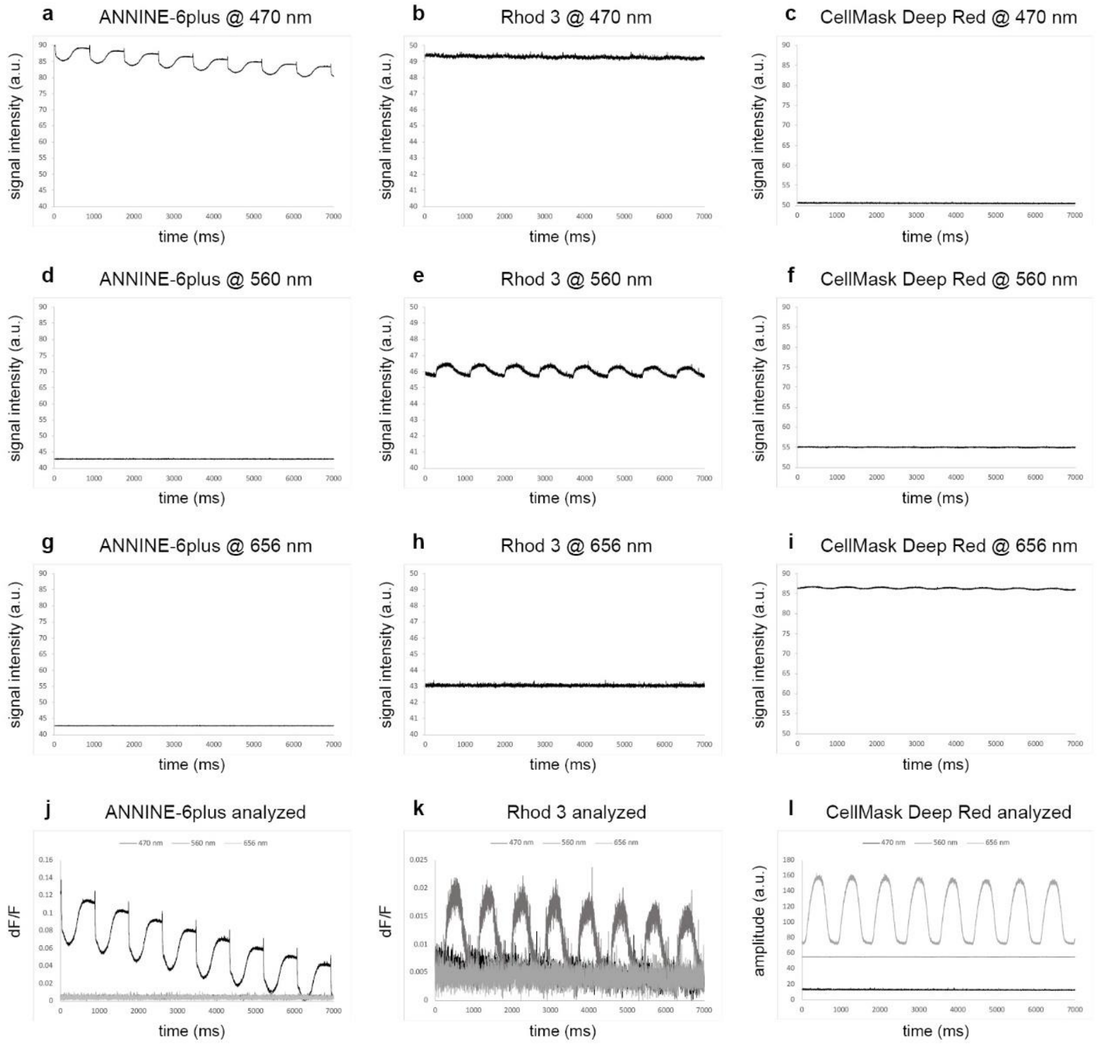

Supplemantary Figure 3: Absence of crosstalk between the different channels. Different wells of hiPSC-CMs were loaded with either ANNINE-6plus, Rhod 3 or CellMask Deep Red. These wells were then exposed to the three different LEDs of the system individually (470nm, 560 nm and 656 nm) to investigate whether a measurable signal occurred in the different channels. Measurable signals where exclusively detected at 470 nm, 560 nm and 656 nm for ANNINE-6plus (a), Rhod 3 (e) and CellMask Deep Red (i), respectively. In contrast, no signals were detected for ANNINE-6plus at 560 nm (d) or 656 nm (g), for Rhod 3 at 470 nm (b) or 656 nm (h), and for CellMask Deep Red at 470 nm (c) or 560 nm (f). To confirm whether the raw signals were indeed not contributing to the analysis of the transients we have performed the first steps of analysis for the dyes: dF/F calculation (ANNINE-6plus and Rhod 3) and MUSCLEMOTION analysis (CellMask Deep Red). Again, the only relevant contribution to the signal was originating from the 470 nm, 560 nm and 656 nm wavelength for ANNINE-6plus (j), Rhod 3 (k) and CellMask Deep Red (l), respectively.

Supplementary Table 1: Overview of all probability scores in the hypothesis table per drug per concentration.

Drug	Concentration (µM)	Contractility+ (cAMP)	Contractility+ (calcium)	Contractility+ (myosin)	Contractility- (calcium)	Contractility- (toxicity)	No change
levosimendan	Vehicle	2.38	3.13	4.64	2.19	3.01	**5.85**
	0.01	1.86	3.31	3.73	1.9	3.18	**4.51**
	0.03	1.94	3.31	4.79	1.8	3.34	**5.47**
	0.1	2.82	3.59	4.53	2.12	2.85	**5.21**
	0.3	2.28	2.73	4	2.82	3.34	**4.9**
	1	1.97	3.29	3.48	2.44	3.38	**4.95**
omecamtiv mecarbil	Vehicle	2.88	3.04	3.9	2.52	2.64	**4.78**
	0.01	2.1	2.6	3.94	2.24	3.24	**4.4**
	0.03	2.4	2.76	**3.73**	2.18	2.49	3.68
	0.1	2.48	2.71	3.1	2.14	2.22	**3.4**
	0.3	4.12	3.46	**4.54**	3.27	2.63	4.18
	1	2.84	3.18	**4.25**	2.01	2.19	3.32
doxorubicin	Vehicle	2.54	3.15	4.29	2.48	3.12	**5.69**
	1	4.07	3.34	4.37	3.47	3.17	**5.38**
	3	3.42	3.37	3.9	2.3	2.52	**4.74**
	10	3.06	2.34	3.66	3.35	3.59	**4.94**
	30	2.09	2.17	2.31	3.62	**4.47**	3.76
	100	0.22	0.18	0.17	0.58	**0.87**	0.19
verapamil	Vehicle	2.74	3.45	4.55	2.46	3.17	**5.69**
	0.01	3.06	2.99	4.16	4.09	4.01	**4.83**
	0.03	3.87	2.27	3.18	**5.66**	4.33	3.27
	0.1	3.63	1.77	2.41	**5.8**	3.7	1.82
	0.3	2.84	1.75	1.52	**4.56**	2.8	1.22
	1	1.42	1.07	0.67	**2.23**	1.17	0.67
aspirin	Vehicle	2.16	2.98	4.25	1.99	2.8	**5.67**
	10	2.09	3.17	4.78	2.35	3.3	**5.5**
	30	2.61	2.76	4.81	2.66	2.86	**5.3**
	100	2.57	3.03	4.95	2.09	2.62	**5.53**
	300	2.22	2.65	4.22	2.79	3.13	**5.07**
	1000	2.95	3.08	4.64	3.1	3.08	**5.27**
captopril	Vehicle	2.62	3.35	3.79	2.66	3.41	**4.86**
	1	2.1	3.26	3.44	1.57	2.82	**4.73**
	3	2.28	3.71	4.85	1.25	2.82	**5.84**
	10	2.3	3.25	3.55	1.83	3.11	**4.81**
	30	2.83	3.61	4	2.22	3.41	**4.77**
	100	2.29	3.31	4.48	1.67	2.93	**5.55**
sunitinib	Vehicle	2.56	3.15	4.18	2.3	3.03	**5.67**
	0.1	1.06	2.72	4.45	1.95	3.3	**4.72**
	0.3	1.15	2.49	3.81	1.87	3.01	**4.13**
	1	1.69	2.28	3.04	2.45	**3.74**	3.65
	3	1.88	1.99	2.62	3.17	**4.38**	3.29
	10	2.69	1.46	2.16	5.02	**5.43**	2.35
atenolol	Vehicle	3	3.45	4.66	2.64	3.1	**5.39**
	0.1	2.98	3.05	**5.12**	2.42	2.84	5.03
	0.3	2.86	2.77	3.97	2.99	3.5	**4.13**
	1	3.03	3.24	4.84	2.89	3.69	**5.06**
	3	3.61	3.31	3.84	3.07	3.31	**4.24**
	10	3.46	3.53	4.96	2.71	3.23	**5.07**
pimobendan	Vehicle	1.94	2.96	4.95	1.83	2.97	**7.01**
	1	2.16	2.89	4.2	1.45	2.68	**5.57**
	3	2.34	3.24	4.52	1.92	2.54	**6.35**
	10	1.95	3.1	4.31	1.48	2.54	**6.06**
	30	2.14	2.91	4.54	1.85	2.36	**4.7**
	100	1.92	2.98	3.29	1.26	1.96	**5.05**
forskolin	Vehicle	2.22	2.89	4.39	2.33	3.08	**5.74**
	0.1	**5.04**	3.74	4.3	3.43	2.77	4.79
	0.3	**6.05**	3.95	3.11	4.33	2.69	3.15
	1	**6.61**	3.87	2.13	5.28	2.86	2.06
	3	**6.63**	3.77	2.45	4.8	2.89	2.51
	10	**6.61**	3.97	2.35	4.45	2.43	1.98
epinephrine	Vehicle	2.11	3.23	5.04	1.88	2.95	**6.7**
	0.01	3.45	3.51	3.3	2.85	3.29	**4.4**
	0.03	4.48	3.84	4.88	3.16	2.66	**5.12**
	0.1	**5.36**	4.17	3.99	4.2	2.74	4.42
	0.3	**4.56**	3.13	2.83	3.42	2.11	3
	1	**5.7**	3.98	3.85	4.48	2.86	4.21
ouabain	Vehicle	2.46	3.05	4.08	2.32	2.98	**5.19**
	0.03	2.66	3.34	4.3	3	2.81	**4.53**
	0.1	2.89	3.65	3.9	2.08	2.43	**4.65**
	0.3	2.45	3.13	4.01	2.34	3.25	**4.88**
	1	2.48	**3.07**	2.33	1.87	2.58	2.56
	3	1.63	**1.7**	0.88	1.4	1.47	0.83

Supplementary Table 2: Effects assigned by the hypothesis based model.

Drug	Epinephrine		MOA: Co^{+}_{cAMP}	
	ALL	AP only	Ca only	Co only
Vehicle	$Co^{=}$	$Co^{=}$ / Co^{+}_{Myosin}	$Co^{=}$	$Co^{=}$
0.01 µM	$Co^{=}$	Co^{+}_{cAMP}	$Co^{=}$	$Co^{=}$
0.03 µM	$Co^{=}$	$Co^{=}$ / Co^{+}_{Myosin}	$Co^{=}$	$Co^{=}$
0.1 µM	Co^{+}_{cAMP}	Co^{+}_{cAMP}	Co^{+}_{Myosin}	$Co^{=}$
0.3 µM	Co^{+}_{cAMP}	Co^{+}_{cAMP}	Co^{+}_{cAMP}	$Co^{=}$
1 µM	Co^{+}_{cAMP}	Co^{+}_{cAMP}	Co^{+}_{cAMP}	$Co^{=}$

Drug	Forskolin		MOA: Co^{+}_{cAMP}	
	ALL	AP only	Ca only	Co only
Vehicle	$Co^{=}$	$Co^{=}$ / Co^{+}_{Myosin}	$Co^{=}$	$Co^{=}$
0.1 µM	$Co^{=}$	$Co^{=}$ / Co^{+}_{Myosin}	$Co^{=}$	Co^{+}_{cAMP}
0.3 µM	Co^{+}_{cAMP}	Co^{+}_{cAMP}	Co^{+}_{cAMP}	Co^{+}_{cAMP}
1 µM	Co^{+}_{cAMP}	Co^{+}_{cAMP}	Co^{+}_{cAMP}	Co^{+}_{cAMP}
3 µM	Co^{+}_{cAMP}	Co^{+}_{cAMP}	Co^{+}_{cAMP}	Co^{+}_{cAMP}
10 µM	Co^{+}_{cAMP}	Co^{+}_{cAMP}	Co^{+}_{cAMP}	Co^{+}_{cAMP}

Drug	Levosimendan		MOA: Co^{+}_{cAMP}	
	ALL	AP only	Ca only	Co only
Vehicle	$Co^{=}$	$Co^{=}$ / Co^{+}_{Myosin}	$Co^{=}$	$Co^{=}$
0.01 µM	$Co^{=}$	$Co^{=}$ / Co^{+}_{Myosin}	Co^{+}_{Ca}	$Co^{=}$
0.03 µM	$Co^{=}$	$Co^{=}$ / Co^{+}_{Myosin}	$Co^{=}$	$Co^{=}$
0.1 µM	$Co^{=}$	$Co^{=}$ / Co^{+}_{Myosin}	$Co^{=}$	$Co^{=}$
0.3 µM	$Co^{=}$	Co^{-}_{Tox}	$Co^{=}$	$Co^{=}$
1 µM	$Co^{=}$	$Co^{=}$ / Co^{+}_{Myosin}	$Co^{=}$	$Co^{=}$

Drug	Verapamil		MOA: Co^{-}_{Ca}	
	ALL	AP only	Ca only	Co only
Vehicle	$Co^{=}$	$Co^{=}$ / Co^{+}_{Myosin}	$Co^{=}$	$Co^{=}$
0.01 µM	$Co^{=}$	$Co^{=}$ / Co^{+}_{Myosin}	Co^{-}_{Ca}	Co^{-}_{Tox}
0.03 µM	Co^{-}_{Ca}	Co^{-}_{Ca}	Co^{-}_{Ca}	Co^{-}_{Ca}
0.1 µM	Co^{-}_{Ca}	Co^{-}_{Ca}	Co^{-}_{Ca}	Co^{-}_{Ca}
0.3 µM	Co^{-}_{Ca}	Co^{-}_{Ca}	Co^{-}_{Ca}	-
1 µM	Co^{-}_{Ca}	Co^{-}_{Ca}	Co^{-}_{Ca}	-

Drug	Doxorubicin		MOA: Co^{-}_{Tox}	
	ALL	AP only	Ca only	Co only
Vehicle	$Co^{=}$	$Co^{=}$ / Co^{+}_{Myosin}	$Co^{=}$	$Co^{=}$
1 µM	$Co^{=}$	$Co^{=}$ / Co^{+}_{Myosin}	Co^{+}_{Myosin}	$Co^{=}$
3 µM	$Co^{=}$	Co^{+}_{cAMP}	$Co^{=}$	$Co^{=}$
10 µM	$Co^{=}$	Co^{-}_{Ca}	Co^{+}_{Myosin}	$Co^{=}$
30 µM	Co^{-}_{Tox}	Co^{-}_{Tox}	Co^{-}_{Tox}	$Co^{=}$
100 µM	Co^{-}_{Tox}	Co^{-}_{Tox}	-	Co^{-}_{Ca}

Drug	Sunitinib		MOA: Co^{-}_{Tox}	
	ALL	AP only	Ca only	Co only
Vehicle	$Co^{=}$	$Co^{=}$ / Co^{+}_{Myosin}	$Co^{=}$	$Co^{=}$
0.1 µM	$Co^{=}$	Co^{-}_{Tox}	$Co^{=}$	Co^{+}_{Myosin} / Co^{+}_{Ca}
0.3 µM	$Co^{=}$	Co^{-}_{Tox}	$Co^{=}$	Co^{+}_{Myosin} / Co^{+}_{Ca}
1 µM	$Co^{=}$	Co^{-}_{Tox}	$Co^{=}$	$Co^{=}$
3 µM	Co^{-}_{Tox}	Co^{-}_{Tox}	$Co^{=}$	Co^{-}_{Ca}
10 µM	Co^{-}_{Tox}	Co^{-}_{Tox}	Co^{-}_{Ca}	Co^{-}_{Ca}

Drug	Pimobendan		MOA: Co^{+}_{cAMP}	
	ALL	AP only	Ca only	Co only
Vehicle	$Co^{=}$	$Co^{=}$ / Co^{+}_{Myosin}	$Co^{=}$	$Co^{=}$
1 µM	$Co^{=}$	$Co^{=}$ / Co^{+}_{Myosin}	$Co^{=}$	$Co^{=}$
3 µM	$Co^{=}$	$Co^{=}$ / Co^{+}_{Myosin}	$Co^{=}$	$Co^{=}$
10 µM	$Co^{=}$	$Co^{=}$ / Co^{+}_{Myosin}	$Co^{=}$	$Co^{=}$
30 µM	$Co^{=}$	$Co^{=}$ / Co^{+}_{Myosin}	$Co^{=}$	Co^{+}_{Myosin} / Co^{+}_{Ca}
100 µM	$Co^{=}$	$Co^{=}$ / Co^{+}_{Myosin}	$Co^{=}$	$Co^{=}$

Drug	Aspirin	MOA:	MOA: $Co^{=}$	
	ALL	AP only	Ca only	Co only
Vehicle	$Co^{=}$	$Co^{=}$ / Co^{+}_{Myosin}	$Co^{=}$	$Co^{=}$
10 µM	$Co^{=}$	$Co^{=}$ / Co^{+}_{Myosin}	$Co^{=}$	$Co^{=}$
30 µM	$Co^{=}$	$Co^{=}$ / Co^{+}_{Myosin}	$Co^{=}$	$Co^{=}$
100 µM	$Co^{=}$	$Co^{=}$ / Co^{+}_{Myosin}	$Co^{=}$	$Co^{=}$
300 µM	$Co^{=}$	$Co^{=}$ / Co^{+}_{Myosin}	Co^{-}_{Ca}	$Co^{=}$
1000 µM	$Co^{=}$	$Co^{=}$ / Co^{+}_{Myosin}	Co^{+}_{Myosin}	$Co^{=}$

Drug	Atenolol		MOA: $Co^{=}$	
	ALL	AP only	Ca only	Co only
Vehicle	$Co^{=}$	$Co^{=}$ / Co^{+}_{Myosin}	$Co^{=}$	$Co^{=}$
0.1 µM	$Co^{=}$	$Co^{=}$ / Co^{+}_{Myosin}	$Co^{=}$	Co^{+}_{Myosin} / Co^{+}_{Ca}
0.3 µM	Co^{+}_{Myosin}	$Co^{=}$ / Co^{+}_{Myosin}	$Co^{=}$	Co^{+}_{Myosin} / Co^{+}_{Ca}
1 µM	$Co^{=}$	$Co^{=}$ / Co^{+}_{Myosin}	$Co^{=}$	Co^{+}_{Myosin} / Co^{+}_{Ca}
3 µM	$Co^{=}$	$Co^{=}$ / Co^{+}_{Myosin}	Co^{+}_{cAMP}	Co^{+}_{cAMP}
10 µM	$Co^{=}$	$Co^{=}$ / Co^{+}_{Myosin}	$Co^{=}$	$Co^{=}$

Drug	Captopril		MOA: $Co^{=}$	
	ALL	AP only	Ca only	Co only
Vehicle	$Co^{=}$	$Co^{=}$ / Co^{+}_{Myosin}	$Co^{=}$	$Co^{=}$
1 µM	$Co^{=}$	$Co^{=}$ / Co^{+}_{Myosin}	$Co^{=}$	$Co^{=}$
3 µM	$Co^{=}$	$Co^{=}$ / Co^{+}_{Myosin}	$Co^{=}$	$Co^{=}$
10 µM	$Co^{=}$	$Co^{=}$ / Co^{+}_{Myosin}	$Co^{=}$	$Co^{=}$
30 µM	$Co^{=}$	$Co^{=}$ / Co^{+}_{Myosin}	$Co^{=}$	$Co^{=}$
100 µM	$Co^{=}$	$Co^{=}$ / Co^{+}_{Myosin}	$Co^{=}$	$Co^{=}$

Drug	Omecamtiv Mecarbil		MOA: Co^{+}_{Myosin}	
	ALL	AP only	Ca only	Co only
Vehicle	$Co^{=}$	$Co^{=}$ / Co^{+}_{Myosin}	$Co^{=}$	$Co^{=}$
0.01 µM	$Co^{=}$	$Co^{=}$ / Co^{+}_{Myosin}	$Co^{=}$	$Co^{=}$
0.03 µM	Co^{+}_{Myosin}	$Co^{=}$ / Co^{+}_{Myosin}	Co^{+}_{Myosin}	Co^{+}_{Myosin} / Co^{+}_{Ca}
0.1 µM	$Co^{=}$	$Co^{=}$ / Co^{+}_{Myosin}	Co^{+}_{Myosin}	$Co^{=}$
0.3 µM	Co^{+}_{Myosin}	Co^{+}_{cAMP}	Co^{+}_{Myosin}	Co^{+}_{Myosin} / Co^{+}_{Ca}
1 µM	Co^{+}_{Myosin}	$Co^{=}$ / Co^{+}_{Myosin}	Co^{+}_{Myosin}	Co^{+}_{Myosin} / Co^{+}_{Ca}

Drug	Ouabain		MOA: Co^{+}_{Ca}	
	ALL	AP only	Ca only	Co only
Vehicle	$Co^{=}$	$Co^{=}$ / Co^{+}_{Myosin}	$Co^{=}$	$Co^{=}$
0.03 µM	$Co^{=}$	$Co^{=}$ / Co^{+}_{Myosin}	$Co^{=}$	$Co^{=}$
0.1 µM	$Co^{=}$	$Co^{=}$ / Co^{+}_{Myosin}	$Co^{=}$	$Co^{=}$
0.3 µM	$Co^{=}$	Co^{-}_{Tox}	$Co^{=}$	$Co^{=}$
1 µM	Co^{+}_{Ca}	Co^{+}_{Ca}	Co^{+}_{Ca}	$Co^{=}$
3 µM	Co^{+}_{Ca}	Co^{+}_{Ca}	Co^{-}_{Tox}	Co^{+}_{Ca}

Supplementary Table 3: A comparison of the outcome of the hypothesis-based algorithm and traditional statistics. P-values were calculated using a Dunnett's test after one-way ANOVA. Source data are provided as a Source Data file.

	Concentration (μM)	Algorithm output	Contraction amplitude	Calcium amplitude	Contraction time	Relaxation time	Calcium time to peak	Calcium decay time	APD90	Action potenial amplitude	Triangulation	AP[Trise]
levosimendan	Vehicle	No change	--	--	--	--	--	--	--	--	--	--
	0.01	No change	p<0.05									
	0.03	No change										
	0.1	No change	p<0.01									
	0.3	No change										
	1	No change										
omecamtiv mecarbil	Vehicle	No change	--	--	--	--	--	--	--	--	--	--
	0.01	No change										p<0.01
	0.03	Contractility+ (myosin)										
	0.1	No change										
	0.3	Contractility+ (myosin)										
	1	Contractility+ (myosin)	p<0.01		p<0.01							
doxorubicin	Vehicle	No change	--	--	--	--	--	--	--	--	--	--
	1	No change					p<0.05					
	3	No change					p<0.05					
	10	No change							p<0.001			
	30	Contractility- (toxicity)				p<0.01			p<0.0001	p<0.001		
	100	Contractility- (toxicity)				NA	NA	NA	p<0.0001	p<0.0001		
verapamil	Vehicle	No change	--	--	--	--	--	--	--	--	--	--
	0.01	No change	p<0.05								p<0.05	
	0.03	Contractility- (calcium)	p<0.01			p<0.0001			p<0.05	p<0.01	p<0.0001	
	0.1	Contractility- (calcium)	p<0.01		p<0.01	p<0.0001		p<0.0001	p<0.0001	p<0.0001	p<0.0001	
	0.3	Contractility- (calcium)	NA	NA	NA	p<0.0001		p<0.0001	p<0.0001	p<0.0001	p<0.0001	
	1	Contractility- (calcium)	NA	NA	NA	p<0.0001		p<0.001	p<0.0001	p<0.0001	p<0.0001	
aspirin	Vehicle	No change	--	--	--	--	--	--	--	--	--	--
	10	No change										
	30	No change										
	100	No change										
	300	No change										
	1000	No change										
captopril	Vehicle	No change	--	--	--	--	--	--	--	--	--	--
	1	No change	p<0.05			p<0.01			p<0.05			
	3	No change										
	10	No change										
	30	No change										
	100	No change										
sunitinib	Vehicle	No change	--	--	--	--	--	--	--	--	--	--
	0.1	No change										
	0.3	No change										
	1	Contractility- (toxicity)								p<0.01	p<0.05	
	3	Contractility- (toxicity)				p<0.01				p<0.0001	p<0.05	
	10	Contractility- (toxicity)				p<0.0001		p<0.0001	p<0.0001	p<0.01		

atenolol	Vehicle	No change	--	--	--	--	--	--	--	--	--	--
	0.1	Contractility+ (myosin)									p<0.05	
	0.3	No change									p<0.05	p<0.01
	1	No change	p<0.01									p<0.05
	3	No change										
	10	No change										
pimobendan	Vehicle	No change	--	--	--	--	--	--	--	--	--	--
	1	No change										
	3	No change										
	10	No change										
	30	No change										
	100	No change										
forskolin	Vehicle	No change	--	--	--	--	--	--	--	--	--	--
	0.1	Contractility+ (cAMP)			p<0.01			p<0.01			p<0.01	
	0.3	Contractility+ (cAMP)			p<0.05	p<0.05		p<0.001			p<0.0001	p<0.01
	1	Contractility+ (cAMP)			p<0.001			p<0.0001		p<0.001	p<0.0001	p<0.001
	3	Contractility+ (cAMP)			p<0.0001			p<0.0001			p<0.0001	p<0.001
	10	Contractility+ (cAMP)	p<0.05		p<0.001	p<0.05	p<0.05	p<0.0001		p<0.01	p<0.0001	p<0.0001
epinephrine	Vehicle	No change	--	--	--	--	--	--	--	--	--	--
	0.01	No change										
	0.03	No change									p<0.05	
	0.1	Contractility+ (cAMP)			p<0.05			p<0.01		p<0.05	p<0.0001	
	0.3	Contractility+ (cAMP)			p<0.01			p<0.01			p<0.0001	
	1	Contractility+ (cAMP)			p<0.001			p<0.0001			p<0.0001	
ouabain	Vehicle	No change	--	--	--	--	--	--	--	--	--	--
	0.03	No change										
	0.1	No change										
	0.3	No change		p<0.05					p<0.05			
	1	Contractility+ (calcium)								p<0.05		p<0.01
	3	Contractility+ (calcium)		p<0.0001						p<0.001		p<0.01
